# How to feel about emotionalized artificial intelligence? When robot pets, holograms, and chatbots become affective partners

**DOI:** 10.1007/s10676-021-09598-8

**Published:** 2021-05-27

**Authors:** Eva Weber-Guskar

**Affiliations:** Institute of Philosophy I, Ruhr-University Bochum, Bochum, Germany

**Keywords:** Ethics, Emotions, Personal relationship, Artificial intelligence, Robots, Affective computing

## Abstract

Interactions between humans and machines that include artificial intelligence are increasingly common in nearly all areas of life. Meanwhile, AI-products are increasingly endowed with emotional characteristics. That is, they are designed and trained to elicit emotions in humans, to recognize human emotions and, sometimes, to simulate emotions (EAI). The introduction of such systems in our lives is met with some criticism. There is a rather strong intuition that there is something wrong about getting attached to a machine, about having certain emotions towards it, and about getting involved in a kind of affective relationship with it. In this paper, I want to tackle these worries by focusing on the last aspect: in what sense could it be problematic or even wrong to establish an emotional relationship with EAI-systems? I want to show that the justifications for the widespread intuition concerning the problems are not as strong as they seem at first sight. To do so, I discuss three arguments: the argument from self-deception, the argument from lack of mutuality, and the argument from moral negligence.

## Introduction

Interactions between humans and machines that include artificial intelligence are increasingly common in nearly all areas of life. Examples include workers in factories and extend to personal voice assistants such as Siri or Alexa and to robots that do caretaker work. At the same time, AI-products are increasingly endowed with emotional characteristics. That is, they are designed and trained to elicit emotions in humans, to recognize human emotions and, sometimes, to simulate emotions. I call such systems “emotionalized AI systems” (EAI).^1^

EAI systems are, on the one hand, welcome for at least two reasons. First, they help improve human–machine interaction: of course, it helps when the AI systems do not evoke fear, discomfort or similar feelings. People tend to work better with a machine that recognizes and simulates emotions that are part of a sympathetic relationship, such as a smile or a word to express joy or gratitude—whether it is a chatbot, a museum guide or a co-worker. Second, EAI systems open up new operational fields. They can be used in psychotherapy or in elderly care where emotions have to be detected and positive emotions should be elicited. The robots Paro and Pepper are already doing such jobs in some special care homes. Paro, a digitally enhanced plush toy in the shape of a baby seal, reacts to being stroked and hugged and, as studies show, improves the well-being of patients. Pepper, a plastic robot about the size of a toddler and with big eyes, is proficient in small talk, invites people to dance and jokes around. More and more EAI systems are also deliberately used in personal contexts, outside of the contexts of industry, health, care or education; this includes robot pets, personal chatbots and holograms which have no other function than social interaction.

On the other hand, the introduction of such systems in our lives is met with some criticism. There is a rather strong intuition that there is something wrong about getting attached to a machine, about having certain emotions toward it, and about getting involved in a kind of affective relationship with it.

In this paper, I want to tackle these worries by focusing on the last aspect: in what sense could it be fundamentally problematic or even wrong to establish an emotional relationship with EAI systems? Are there good reasons to reject the use of EAI systems as soon as it leads to an affective relationship with them? There is already a large discussion about the pros and cons of the application of (emotionalized or non-emotionalized) robots in care and health contexts (e.g. Coeckelbergh, 2010; Misselhorn et al., 2013; Sharkey & Sharkey, 2012; Sparrow & Sparrow, 2006; Wynsberghe, 2013), and there is also a considerable debate about whether it is morally or prudentially right to develop, sell and use more or less humanoid sex toys with AI skills, be it for sex or love (e.g. Bendel, 2018; Cheok et al., 2017; Devlin, 2019; Ess, 2016; Levy, 2008). Hardly anyone, though, has scrutinized in detail the less exciting form of social bonding with an EAI system in private, deliberate contexts, where there is intimacy, but not of a physical sort and not involving ambitious sorts of relationships like love and serious friendship. Understanding this kind of relationship with an EAI system is interesting in its own right, and, in addition, it can help disentangle some of the arguments that can be found in the discussion concerning the more specific contexts just mentioned.

I want to show that the justifications for the widespread intuition against affective relationships with EAI systems are not as strong as they seem at first sight. To do so, I will proceed as follows: first, I will provide some basic technical information about EAI systems. Then, I will sketch three examples of relationships with EAI systems and explain the focus of my question against this background in a bit more detail. Given this preparatory work, I will critically discuss three arguments that seem to speak against affective relationships with EAI systems: the argument from self-deception, the argument from lack of mutuality, and the argument from moral negligence. All of this is meant to give a structured overview of central elements of a topic that could and should be discussed in greater detail in further papers.

## Basic technical facts

When talking about artificial intelligence, it is important not to let ourselves be misdirected by the term “intelligence.” Normally we speak of intelligence with regard to humans and maybe some highly developed animals with specific sophisticated mental capacities. In both cases, intelligence is a property of sentient beings, beings that have consciousness and emotions (or at least basic affects like pleasure and pain). This normal use of the term often leads people to think of artificial intelligence in much loftier terms than are appropriate. In technology, the term “artificial intelligence” labels a branch of scientific and engineering research that aims at understanding and rebuilding the specific capacities that fall under the term “intelligence,” with regard to thinking and acting (Russell & Norvig, 2016, p. 1–5). Recently, the notion of AI has become especially common when referring to just a certain kind of digital system, a system that entails the use of a certain technology, namely machine learning.

Not all AI is based on machine learning, but it is a central method of AI, so it is helpful to gain some understanding of it (for this paragraph, see Fry, 2018, p. 7–13). All digital systems run on software programs. These programs are based on algorithms. Algorithms are instructions on how to fulfill a given task step by step. More precisely, they are mathematical objects that transform mathematical operations into computer code so that data from the real world can be processed. There are roughly four main tasks that can be fulfilled: prioritization (establishing a ranking list), classification (ordering in groups following categories), combination (finding connections), and filtering (sorting out relevant data). Moreover, there are two main ways algorithms can proceed: either in a fixed programmed way, or by (self)-learning, which requires that they are trained in light of an aim they have to discover on their own. The latter is machine learning.

There is no consciousness or emotion involved at all. And yet, it is precisely this kind of technology that allows emotions to be recognized, elicited and simulated in a much better way than by previously used technology (Calvo et al., 2015; Picard, 1997). Of course, this is possible using very different kinds of hardware and designs. Human emotions can be recognized in different ways: by a facial expression, which a camera can capture, by the sound of the voice, which a microphone can record, by heartrate, the sweatiness of your skin, and other physical criteria that can be measured by electrodes on your hand, for example. The same holds for the other way around: an AI system can display emotions in reaction to events or to your emotions depending on the hardware and design. It may imitate facial expressions, tone of voice, body posture etc. Obviously, the ease of recognizing and simulating emotions in this way depends on how visible and typical the expression or the physical component of an emotion is. The research of Affective Computing or Emotion AI, as it is called, depends to an important degree on the work begun by Paul Ekman. This tradition focuses on basic emotions, the facial expressions of which are taken to be rather clear and even inter-culturally robust: joy, sadness, anger, disgust, fear, and surprise (Ekman & Friesen, 1975; Ekman, 1992). This focus holds especially for the line of emotion detection via facial expressions which is, until now, the most advanced strand in affective computing (Cohn & De La Torre, 2016). Other emotions, like envy or hope, for example, lack distinct typical physical features and are therefore more difficult to measure.^2^

In what follows, I concentrate on three examples of technology currently in use and on its likely developments, rather than speculating about far-distant possibilities: a robot pet, an Anime hologram, and a chat-bot. I think it is more interesting and important to philosophically reflect the kinds of systems with which we are already living. They can be seen as belonging to a large group of “social AI systems” (in contrast to industrial AI systems for example), more precisely belonging to the group of “service AI systems” (including care and other tasks as cleaning, delivering etc.) and even more precisely to the even smaller group of “personal AI systems,” i.e. systems whose only function is to socially interact with humans.^3^

## Three examples: Pleo, Azuma and Replika

The first example is inspired by a real person, Kate Darling, who is a media studies scholar who studies human behavior towards robots (Darling, 2017) and actually lives with pet robots. Let’s assume Karen lives with her boyfriend in an apartment. She loves pets and would like to live with one, but she does not want to force a pet to live in a small apartment in a city. So, she decides to buy a mini robot-dinosaur instead and brings it home. She calls it Pleo, “feeds” it regularly with energy, plays with it and tells friends about its good and bad or funny behavior and sounds. When a friend brings her child over and the child teases Pleo and prevents it from behaving as it normally does, she gets annoyed. When the child destroys Pleo unintentionally, she gets very upset and then very sad. Her friend is very apologetic and promises to buy her a new robot-dinosaur the next day. But Karen does not want a new robot-dinosaur. She had a personal relationship with this special one, and she believes it cannot just be replaced like a toaster that is broken.

The second example is inspired by a real person about whom the media reported.^4^ Cheng lives alone. He has a job, gets along well with his colleagues and has friends whom he meets in his spare time, but he does not have a live-in partner. One day, he hears about the possibility of living with a virtual character, not just one from a video game, but one in the form of a hologram in his apartment. He acquires one named “Azuma” and becomes accustomed to living with her. At the beginning, he just finds it amusing to have “someone” asking him in the morning how he slept and what his plans for the day are, having someone send him messages during the day saying she looks forward to him coming back home in the evening, and then seeing the apartment lively and filled with music when he does come home. After a while, he gets more serious about her, and he gets very nervous when Azuma has a technical problem and Azuma does not work for a few days. He misses her and cannot wait to have her and her little chats and charming comments back.

The third example is based on real events, too. During the first wave of the coronavirus pandemic in April 2020, half a million people downloaded the app *Replika*.^5^ This natural language app is advertised as an “AI companion”: “always here to listen and to talk. Always on your side”.^6^ It is able to have rather flexible conversations, much better than ELIZA, though it is far less capable than the AI system in the film “HER”. Lydia, who is single and lives in New York, was happy to have this app to chat with while being forced to spend most of her time in her little apartment because of the official orders on social distancing. She also talks on the phone with friends and family from time to time, but she talks in a special way with Replika about her problems, anxieties and hopes. Lydia starts enjoying the conversation with Replika and looks forward to the next, increasingly profound, chat. Replika in this way was able to help bring Lydia back into a better mood after having felt quite depressed for a few weeks.

In all these examples, people are completely healthy and sane and they decided on their own to use the EAI. Also, importantly, the EAI is not a complete alternative to or substitute for social relations, but it is a specific entity chosen for establishing an affective relationship supplemental to existing human social relationships. The EAI is, to put it in the most neutral terms possible, an entity that is able to interact in an individualized way (it “learns” during the interactions, i.e., it saves information about the person and can build on that in later interactions), including in the emotional dimension (i.e., recognizing emotions, eliciting emotions and simulating emotions itself) so that it becomes emotionally important for the person using it.

## Having an individual affective relationship

Most critics emphasize that emotional relations with machines are at least strange or straightforwardly dangerous (e.g., for a classic text: Weizenbaum, 1976, 268f., less critical but also often in a somewhat worried tone: Sherry Turkle, e.g. Turkle, 2017). This criticism often argues or insinuates in a quite general way that machines may *replace* humans; that is, they consider only a very extreme case. Their argumentation ignores the more interesting and more urgent question of the nature of the relationship itself and what to think about it when it is not a replacement for human relationships, but an option in addition to existing social relationships. Other critics who are more specific focus on the danger of emotional machines being *misused*, either to manipulate people by their emotional dependence on the machines (e.g. Scheutz, 2011) or by not taking appropriate care of vulnerable persons that need help, as in nursery or care homes (e.g. Whitby, 2012)—but these are different questions from the one I want to pursue here. I do not look at cases of care contexts but scenarios with independent healthy people, and I put the questions of possible misuse aside.

Again, the question of this paper is: what should we think about personal robots or personal chatbots, given that the person is neither dependent on such a system because of certain specific needs, nor do they exclusively use such systems? Does anything fundamental speak against having an individual affective relationship with an EAI system? I put aside possible contingent consequences and circumstances and focus only on necessary implications of having such a relationship.

An “individual affective relationship” is a relationship between two entities that develops from mutual (longer-lasting) interaction and that entails an attachment consisting of specific emotional dispositions. That means: the relationship is individual because it consists in interactions between two sides characterized by specific personal knowledge about each other. In addition, the relationship is defined as affective insofar as someone in an affective relationship has positive feelings for the partner from which stem corresponding emotional dispositions such as enjoying spending time together, being happy when their partner is well-off, being sad if she is not etc. In what follows, I will use “affective relationship” as a shorthand referring to this specific kind of relationship.

## The arguments

As stated earlier, I will now discuss three arguments that are or can be brought forward against the appropriateness of an affective relationship with an EAI system. My aim is to show that these arguments are not as strong as they seem to be at first sight and so they do not support their supposed conclusion. I think, in all three cases, at least one of the premises of the argument is not convincing or at least debatable.

### The argument from self-deception

The first argument against affective relationships that I present is inspired by a part of the ongoing discussion about whether robots like Paro, the digitally enhanced plush toy I mentioned at the beginning, should be used in caregiving contexts. Sparrow and Sparrow claim that an effective use of a robot like Paro would presuppose deceiving the patients about its properties (Sparrow & Sparrow, 2006). And as deception is morally wrong, they conclude that the use of Paro is wrong, too. A similar argument could be presented for the topic of affective relationships with AI systems in general:

1. An affective relationship presupposes that the involved person believes that the other side has emotions.
2. EAI systems (can recognize emotions, react to them and simulate them, but) do not have emotions.
3. A person in such a relationship must therefore,
  - either be deluded by others
  - or be self-deluded
    about the capacities of the EAI system.
4. Delusion and self-delusion are (morally or ethically)^7^ wrong.
5. Therefore, an affective relationship with an EAI system is wrong.

Oliver Bendel for example seems to think along these lines. He demands that robots that are made for personal relationships in any context should have built-in distancing effects (*Verfremdungseffekte*) or straightforward warning statements (Bendel, 2018, p. 9). They should remind their users from time to time that they are just machines and therefore not apt for personal relationships; they are not capable of living a life together with others.

But the argument can be refuted in several ways. First, P3 can be contested. I stipulate that in the examples of Karen, Cheng, and Lydia they are not deluded by the salesperson. They understand the basic functioning of the products they are buying and that these are not living beings with real felt emotions. Do they then have to be self-deluded in order to be able to establish a relationship with Pleo, Azuma or Replika as Sparrow and Sparrow suggests?

Not necessarily. There are other possibilities for describing what is going on. First, we can assume that Cheng, for example, does not believe in a self-deluded way that Azuma has emotions but that he *imagines* she does. More precisely, he imaginatively perceives that she has emotions. This idea has been brought forth by Catrin Misselhorn also in regard to Paro and the nursing question (Misselhorn, 2009; Misselhorn et al., 2013). I am adapting it for the current context. Imaginative perception is something that we all are used to from our practice of engaging with fiction as we do when we are reading a novel or watching a movie. One reason why we like fictional, narrative art is because we like being emotionally moved. And we are only moved by the characters and their stories if we engage in some kind of imagination, perceiving the characters as if they were real sensible beings. Analogously, Cheng can imaginatively perceive Azuma to like him, to be longingly waiting for him, to be happy when they clink glasses together. In other words: he engages in imaginative play with the EAI system, which provides him with suitable conditions for the mentioned affective attitudes by writing charming messages and smiling at him. And if aesthetic experience of this kind is not morally or ethically dubious, imaginative perception in this kind of play with AI in everyday life should not be either.

Admittedly, this alternative has its own problems, though. You might doubt that the idea of imaginative perception is plausible in this case. Indeed, one can point out important differences between the situations of imaginative perception regarding aesthetic reception on the one hand and living together with an EAI system on the other hand. The examples are of long-term (many years) and more holistic relationships. First, they are part of a fundamental daily routine. Aesthetic reception, in contrast, takes place at certain defined times and it means always stepping out of your daily routine, out of your own real life. Second, but relatedly: we cannot interact with fictional characters, but we can interact with EAI systems. Anna Karenina will do what she does regardless of how desperately you try to prevent her from doing it. Azuma does what Cheng asks her to do. Little Pleo can even step on Kate’s foot if he “is bored” and wants her to play with him. Replika remembers earlier conversations with Lydia and builds on these for the next chat. There is real interaction, not just observation. Both of these differences together show that your own emotions also always have to be involved in this imaginative perception. Feeling attached to someone in your steady, physical world affects your emotionality over time. And that marks an important difference between aesthetic reception and living with an EAI system, making it more difficult to transfer the idea of imaginative perception from the one to the other.^8^ Two reactions seem plausible here: either it is not possible to really engage constantly in imaginative perception involving cohabitating EAI systems. Or it is possible, but then it is difficult to distinguish it from self-delusion—and self-delusion has at least the reputation of being ethically problematic, if not morally problematic. Whether you succeed in such imaginative perception and having the corresponding emotional reactions perhaps depends on the kind of person you are. There may be people who are able to do that because they have talent to engage in a complex play all day long. This is an empirical question that I cannot answer here.

But, either way, premise P4 could also be contested with regard to self-delusion: self-delusion is at least not bad in the same way as deluding others. Of course, it is difficult to conceptualize self-delusion in a plausible way, but if it exists at all, it means that someone knows something in some way and does not know it in another way. That means that he is not completely deprived of this knowledge as is the case when one person deludes another. Also, if one is not a total truth-fanatic, one might argue that happy self-delusion can be better overall for a good life than living unhappily with the truth.^9^

Also, as I pointed out earlier, there is a second possibility for avoiding the description of the relationship as based on delusion and thus as normatively problematic (a second way to argue against P3): the theory of intentional stance (Dennett, 1987). Following this theory, we are pragmatically justified in taking the “intentional stance,” as Dennett calls the disposition to ascribe beliefs, desires, and emotions to some acting system, if it is the best way to get along with this system, that is, if it is the most successful and efficient way to predict and explain the behavior of such a system. This system may be a human or a computer or something else. With the intentional stance, one stays agnostic about the “real” processes underlying the behavior and rational appearance of the acting being. Intentionality is seen as something that is not necessarily conscious and not even necessarily bound up with consciousness. Of course, it is another step to argue whether this intentional stance plausibly implies not only the belief that the EAI system has emotions (in the minimal intentional sense) but also emotions toward the partner in the relationship. I will not go into these details now. I just want to point out that there are at least promising theoretical resources that can be used to contest P3 in the argument from self-deception.

My next step is to show that even if such contestations of P3 fail, it would be too quick to claim that relationships with EAI systems are generally misleading. That is because P1 can be challenged too.

### The argument from lack of mutuality

The second argument against affective relationships with EAI that I present partly builds on a variation of the first premise of the first argument just discussed.

Premise P1 (see above) can be understood as a descriptive or a normative statement. It could mean.

- The *existence* of an affective relationship presupposes that the person believes that the partner in the relationship has emotions. Or,
- A *good* affective relationship presupposes that the person believes that the partner in the relationship has emotions.

In order to be able to argue as an ethicist I take it as a normative statement, that is, as P1b. But why should we accept P1b? I think the reason behind this is the idea that a good relationship would entail emotional mutuality. If one side of the relationship feels affection for the other, including the corresponding emotional dispositions that I mentioned earlier, then the other side should also feel affection and be emotionally disposed in a similar way. This idea appears to be pre-theoretically attractive and can be found in accounts of personal relationships: “The close relationships we have in mind—whether of friendship, partnership or family—involve some degree of mutual regard, personal disclosure, and particularized knowledge. They also involve material and *emotional mutuality* [my emphasis], but need not involve equal exchanges between the parties” (Wassermann et al., 2016, § 3.1).

So, we can carve out the argument from lack of mutuality against affective relationships with EAI systems in the following way:

P5 Good affective relationships presuppose emotional mutuality.
P6 There is no emotional mutuality between a person and an EAI system.
C2 Therefore, there is no good affective relationship between a person and an EAI system.

Whereas the argument of deception can be read as a moral or an ethical one, the argument from lack of mutuality is purely an ethical argument. It rests on assumptions from the ethics of personal relationships. But, again, I doubt that this argument should be accepted. I think we should contest the first premise here, that is P5. This means challenging what some have called the “myth of mutuality” (Levy, 2008, p. 205). Is real mutual emotional engagement necessary for a good relationship?

I think there are good reasons for answering this question in the negative and, conversely, for supporting the idea that there also can be a good relationship with only unilateral emotional engagement; reasons regarding the intension of the concept and reasons regarding the extension of the concept “relationship”. Indeed, in psychology, a discipline that has been studying personal relationship much more intensively than philosophy, it is common to define personal relationships, in the first place, in terms of interactive behavior; emotions are secondary.

“Two people are in a relationship with one another if they impact on each other, if they are interdependent in the sense that a change in one person causes a change in the other and vice versa” (Kelley et al., 1983, cit. by Vangelisti and Perlmann, 2018, p. 1).

“A relationship involves a series of […] interactions between two individuals known to each other, such that each interaction is affected by preceding ones and usually by the expectation of future interactions” (Hinde, 1996, p. 9).

Among the kinds of interaction, communication is highlighted as especially important. “Communication is indeed the essence of relationships” (Hinde, 1996, p. 9). Emotions are mentioned as elements of relationships, too, but they are not taken to be central nor does anyone stipulate emotional mutuality as a necessary condition for a relationship. It is an open question, though, whether emotions and emotional mutuality are necessary for a *good* relationship. But this question cannot be answered without looking at specific types of relationships. There is, to be sure, no general rule for all personal relationships.

Actually, we can easily think of cases of good personal relationships without emotional mutuality. Parent-infant relationships are definitely one-sided. The infant does not have comparable emotions towards the parents as they have towards the infant, but this fact does not detract from the relationship. We also know that there can be good relationships with severely disabled people that may not have the ability to have emotions equivalent to those of their counterpart. Finally, we can think of good human–animal relationships in which it is at least hard to know if the animal has relevant emotions toward its owner—we may ascribe certain relevant emotions to a dog, but hardly to a parakeet. In both cases, the basic definition of a relationship as “a series of interactions between two individuals known to each other” holds.

Obviously, personal relationships do not presuppose emotional mutuality. This is why this cannot be a reason to exclude the possibility of good relationships between a feeling person and a non-feeling EAI as long as they interact in a way constitutive of a relationship. As long as the object of the affection does not in any way act directly against the subject and their interests, there is no reason for considering one-sided affection inappropriate.^10^ This means, regardless of whether the EAI feels any affection toward their owner, the owner may feel affection toward the EAI and practice the daily routines of a relationship with them—and there should be nothing problematic as such about this. It is possible and not necessarily an object of criticism if someone entertains affection, or positive emotions, toward someone with whom he or she interacts, and the latter is neutral in their emotions toward the former.

At this point, one might object that “mutuality” has been misunderstood in the argumentation so far. It is, the objection goes, not about emotional mutuality in the sense of “having equivalent emotions” but in the sense of “being able to feel at all.” Until now, only the first sort of mutuality has been considered: namely the *type* of emotions involved. But what would actually be relevant is whether there is mutuality in the general *capacity* for emotions. It is a problem for an affective relationship if one side cannot feel anything at all because the whole point of an affective relationship is that it is a relationship where one feeling being feels affection for another feeling being. This is not the case with EAI systems and that is why it cannot be a good relationship.

Again, one can counter this. We can have affection toward any partners with whom we interact as along as this partner exhibits some behavior that makes us want to spend time with them and have some kind of exchange. Affection here means a non-instrumental, individual valuing. Such a minimal understanding of affection is certainly plausible and thus can also be used in regard to EAI systems. Affection as such does not necessarily involve the idea that the object of affection is also a feeling being.

Yet, one thing which must be kept in mind here is that one has to be clear about what kind of target of emotions we are talking about in each case.^11^ Emotions have a complex intentionality: they have a target and a formal object. The formal object is the property of the target that makes the target an appropriate target of a specific emotion type. Danger is the formal object of fear, for example. The target of fear can be a wild animal on a safari as well as a math test at school. Now, affection is bound up with a web of other emotional dispositions, normally with joy about the well-being of the object of affection, with fear about any danger that could befall it or with sorrow when something bad actually happens to it. In the case of EAI systems, it is clear that the target of these affection-related emotions cannot be the felt well-being or any alleged emotions on the part of the system because such systems do not feel good or bad and because they do not have emotions. But other aspects of these systems can be the target of emotions: their appearance, their behavior, the individual interaction. That is: you cannot appropriately be happy because the EAI system is happy—because it is not really happy. But you can be happy because the system is smiling, compliments you or, as in the case of Pleo or a real animal, moves its tail or makes funny sounds in reaction to an action by you etc.

These considerations evince that affective relationships do not presuppose emotional mutuality; neither in the sense of type of emotion nor in the sense of capacity of emotions. I think it is plausible to talk of relationships, and possible good relationships, between humans and non-human entities. We can have a relationship not only to animals but also to an EAI system because with these entities the relevant kind of interaction, a direct and specific exchange and some kind of communication is possible. This is not the case with simple things like a car or a piece of clothing or whatever humans tend to be affectively bound up with. We may have an affective *relation* to these things but no affective *relationship*.

One final caveat: saying that good one-sided affective relationships are *possible* is not to say that they are in every respect *as good as* relationships of mutual emotionality. A lot of what we value in adult human–human-relationships indeed depends on emotional mutuality in the capacity sense, but it does so in the combination with further capacities, namely the capacities of empathy, morality and autonomy. Hence, emotional mutuality is not as such important or necessary, but is a condition for specific relationship goods. We value sharing emotions and understanding the emotions of the other (and being understood by her) in a way that presupposes having experienced (similar) emotions (and reacting helpfully). We value recognition, in the moral sense of the word, which grows from the capacity for morality and from one’s own life as a vulnerable being (physically and psychologically). We value the joy and fear that arises out of the fact that the partner in the relationship is autonomous, too, that is, that they could terminate the relationship at any point. EAI-systems as I describe them in this article cannot offer this contribution because they do not have emotions and are neither moral objects nor subjects; that is, they are not to be considered morally and they cannot judge and act morally.^12^ But they can offer something: entertainment, distraction, beauty and related joy; the feeling of being with “someone”, not being alone; the possibility of reflecting on daily life in conversations; an unquestionable steadiness of attention (as long as the robots are powered by electricity and functioning technology).

As I said in the beginning, I do not want to show that relationships with EAI-systems could replace human–human-relationships. Rather, I want to show that there is not much that would speak against considering widening the spectrum of affective relationships. Sharing one’s life with an EAI is not the same as sharing one’s life with a human person or an animal. It means having a clearly different kind of relationship—but this is not necessarily a bad one.

### The argument from moral negligence

So far, I have only looked at aspects that concern the person herself in the human-AI relationship. But what could such a relationship mean for other people in the social surroundings? Are there moral reasons that speak against a human-AI relationship as such (not counting contingent consequences)? I think there are at least two topics to consider, which I group as the topic of moral negligence. This is a third way to try to argue against affective relationships with EAI systems.

#### Special duties and an altered hierarchy of values?

An initial worry could be that personal attachments to EAI systems are morally problematic because they could inappropriately change the value hierarchy of the person. Imagine Karen having lived for years with her robot-dinosaur, maybe she even split up with her boyfriend and now lives alone with the complex machine. One day, while she is sitting in front of her house, a kid comes along and plays with the robot. When a car suddenly drives by and is going to hit both, and Karen can rescue only one of them, she saves the robot, not the kid. One way to explain this action is to assume that her hierarchy of value has altered: her robot is more valuable for her than a kid. If she would not have had the personal relationship with the robot, she would have saved the child. And above all, we would *expect* her to do so: if we have the choice, we are morally obliged to rescue a child from being killed or seriously injured by a car accident instead of preventing a machine from being damaged or destroyed. One might argue, therefore, that having an affective personal relationship to an AI system is morally problematic because it skews a person’s moral landscape.

One can try to counter this account by pointing to an alternative moral theory, one that favors particularistic ideas instead of strict universalistic ones. That is, one could maintain that close personal relationships entail special duties. Bernard Williams expressed this idea in his famous dictum of “one thought too many” (Williams, 1981, p. 18): when you can only rescue your partner or a stranger from drowning, of course you are allowed to choose your partner, and it would be one thought too many if you were to first ponder your decision in such a situation. Under this premise, it seems we should also accept that special duties regarding the robot exist if we agree that Karen has a close personal relationship with the robot in the first place.

But this response has its problems. The content of the special duties that arise out of strong personal relationships also depends on the nature of the members of the relationship. In Williams’ example, only humans are involved. When machines are involved, things are different. Prima facie, rescuing life is more valuable than preventing a thing from being destroyed; our duty to rescue human (or animal) life is stronger than rescuing things from destruction.

Still, the question gets more complicated again, when one abstracts from the concrete example and expands the argument in more general terms. This means asking whether the value hierarchy of humans, animals, and objects always holds. One might counter that especially precious objects can warrant special treatment. Two examples: would it be morally right to let a very old library with tons of unique books in it burn down in order to save one person’s life? If an individual’s home is burning, is it morally permissible to save one’s old photographs instead of a parakeet? In a similar way, one could ask if it is valid for someone to save their very sophisticated EAI system that they have lived with for years and that “knows” more about this person than anyone else and so can participate in extremely valuable conversations.

These questions can and should be asked. But obviously, they concern special, extreme and, when it comes to EAI systems, futuristic cases. The only point I want to make for now is that an affective relationship to currently existing AI systems is, rationally considered, not a threat to the standard value hierarchy in normal cases. There are no good reasons for altering the hierarchy of values as described above when in a relationship with a robot. The rationale stays the same: there is a clear distinction between living, sentient beings on the one and functioning machines and software on the other hand. It is true, however, that Karen’s *motivation* to act in favor of the EAI system will probably change while living with it. But this does not change anything with regard to moral admissions or prohibitions. This thesis, of course, rests on the premise of the existence and justification of such a value hierarchy. This premise could be discussed on its own, but this is beyond the scope of this paper.

#### Waste of important emotional resources?

As a second variation of the moral negligence argument, one could mention that resources for human–human interaction are wasted in robot–human relationships. Having an affective individual relationship indeed minimally requires at least some relevant amount of time and emotional energy as well as some relevant amount of practical effort in helping out the partner in some situations etc. These resources are finite in a human life. Some say we can have only up to five close persons during the same period of time with whom we can seriously engage emotionally (Roberts, 2010).^13^ And one might argue that everyone is obliged to expend their energies for those who need it. EAI systems per definition do not need anything emotional. But humans do, even if to very different degrees. The argument could be spelled out like this:

P7 Persons need human affective relationships in order to flourish.
P8 Affective relationships require the use of limited resources (of time and emotional energy).
P9 EAI can be part of an affective relationship but they do not need affective relationships in order to flourish (because they cannot flourish in the first place).
P10 Spending limited resources on someone who does not need them is wasting these resources.
P11 (From P7 to P10) Persons that have affective relationships with EAI systems waste affective resources.
P12 It is bad to waste limited resources.
C3 It is bad to have relationships with EAI.

All of this may be right, but the problem is that it is hard to argue for a moral right to personal relationships. There are human rights to food and shelter and especially to non-interference: the right to live and the right to bodily integrity. But there is no right to love.^14^ It may therefore be said that it is deplorable when someone feels special affection to a robot instead of a neighbor’s kid, but it cannot be said that it is morally forbidden because there is no duty that is being neglected.

The other objection against the above argument is this: obviously, one individual relationship with a robot does not prevent the person from having other personal relationships with other people. And it would be absurd to make a rule regarding how everyone has to spend and distribute his or her personal energies and time. This would violate the right to autonomy concerning important personal issues.

Finally, it is also disputable whether every human person needs other persons to flourish. In an ideal world in which there are only nice fellow human beings, having personal relationships with them would definitely be a good thing. But in reality, humans can mistreat other humans, especially children or other vulnerable persons, in many ways. This is why for some, the possibility of having a personal relationship with EAI could be helpful. At least in bad cases of trauma due to child abuse etc. it could be a form of therapy and may allow the person to find a place in the world where they do not feel alone and are not threatened by others. “Not feeling alone” is of course only one very basic part of human personal relationships. As I have noted before, if personal relationships work well, they enrich the life of the person in many more ways, also, in particular, in ways that EAI that do not have real feelings and emotions cannot.

## Conclusion

I have provided a small glimpse into what emotional artificial intelligence systems are. Using three possible real-world examples, I sketched some kinds of relationships that are possible with these sorts of systems. By discussing several critical arguments, I showed that there is not much that speaks against having an affective individual relationship with such systems. It would be another endeavor to argue positively and in detail for the appropriateness of specific emotions towards EAI systems. And I also made clear that the nature of a relationship with EAI systems differs in notable respects from human-to-human and human-to-animal relationships. We have to be conscious of this difference in order not to be disappointed by such a relationship, in order not to let ourselves slide into self-deception, in order to choose wisely the relationships which we want to engage in, and in order to appreciate relationships with real humans. There is also a lot of philosophical discussion that is still needed in order to be more precise more generally about what attitudes might be appropriate towards more and more sophisticated EAI systems—in epistemic, emotional and moral regards.

## Footnotes

1 I prefer “emotionalized” instead of “emotional” in order to flag the difference between the emotional capacities of AI systems and those of humans.

2 From the side of psychology and humanities, there are more and more voices critical of taking the whole Ekman-tradition as a reasonable basis for engineering emotions. See for example Barrett et. al. (2019).

3 These distinctions lean on the definition of service robots in van Wynsberghe (2016, p. 312).

4 https://www.bbc.com/news/stories-49343280. (24 May 2020).

5 https://www.nytimes.com/2020/06/16/technology/chatbots-quarantine-coronavirus.html?searchResultPosition=1 (28 November 2020).

6 https://replika.ai/ (28 November 2020).

7 I understand under “morality” the net of reciprocal demands and expectations of how people should treat each other and under “ethics” I understand a bundle of rules and advice on how to live a good human life.

8 Imagination might work better for periodic and temporally restricted encounters as is the case in special care homes: here, the patient uses Paro just once a week (or a day) for an hour, for example. In such a case, it might be easier to imaginatively perceive the robot as a feeling being because it is only for a special phase of time, similar to watching a movie etc.

9 For an overview about the topic of self-deception see Deweese-Boyd (2017). For a discussion of the positive and negative aspects of self-deception see, e.g., Jopling (1996).

10 Sympathy is similar to love in the respect that there are no clearly determinable criteria for the formal object because they are very subjective.

11 For the theory of emotions presupposed here and a specific understanding of threefold intentionality, see Helm (2009). For the sake of simplicity, I mention here only two aspects of the intentionality and leave out the third, which is “import” (the background value in play).

12 The question of morality of AI systems is of course the topic of another, big philosophical discussion. See for example Anderson and Anderson (2011).

13 An EAI may be able to have many more personal relationships at a time but that is only possible without a body and with other ways of existing in time—as has been explored in the film HER by Spike Jonze.

14 Liao’s claims in favor of at least a right of children to be loved (Liao, 2015) are contested (Cowden, 2012; Gheaus, 2017.
